# Scalable Fabrication of MOF Glass Membranes for Gas Separation via Stepwise Phase Transition

**DOI:** 10.1002/advs.78073

**Published:** 2026-09-29

**Authors:** Mao Ye, Yi Yang, Zibo Yang, Yuxiu Sun, De Ao, Fan Zhou, Ying Chen, Chunhui Luo, Ziyao Wang, Wenjia Sun, Yunling Jia, Wengang Huang, Jingwei Hou, Zhi Wang, Zhihua Qiao

**Affiliations:** ^1^ State Key Laboratory of Advanced Separation Membrane Materials Tiangong University Tianjin China; ^2^ School of Textile Science and Engineering Tiangong University Tianjin China; ^3^ School of Chemical Engineering and Technology Tianjin University Tianjin China; ^4^ School of Chemical Engineering The University of Queensland St Lucia QLD Australia; ^5^ Hebei Center for New Inorganic Optoelectronic Nanomaterial Research Handan University Handan Hebei China; ^6^ School of Materials Science and Engineering Inner Mongolia University of Science and Technology Baotou China; ^7^ ARC Centre of Excellence for Green Electrochemical Transformation of Carbon Dioxide Brisbane Australia

**Keywords:** gas separation, MOF glass membrane, salable fabrication

## Abstract

MOF glasses have emerged as promising membrane materials, yet the scalable fabrication of macroscopically continuous and crack‐free MOF glass membranes remains a critical bottleneck that hinders their industrial translation. Here, we report a stepwise phase transition strategy that enables the formation of large‐area MOF glass membranes. By constructing a crystalline‐amorphous composite precursor, this strategy mitigates electrostatic repulsion and leverage graded viscoelastic flow to promote early‐stage interparticle bonding. This approach effectively suppresses cracking and pinhole defect formation during large‐area processing, providing a viable pathway toward scalable glassy membrane manufacturing. Based on this method, a record‐breaking 100 cm^2^ MOF glass membrane exhibiting a CH_4_ permeance of ∼13,000 GPU and CH_4_/N_2_ selectivity of ∼4.4, alongside a validated disc‐type module, has been successfully fabricated. This strategy establishes a scalable route for producing large‐area MOF glass membranes and advances their application in real‐world gas separation scenarios.

## Graphical abstract

Metal‐organic framework (MOF) glass membranes, with their excellent processability and permanent pore structure, have great potential for gas separation applications. However, during large‐scale fabrication, defects and obvious cracks are prone to occur due to the limitations of powder processing. Based on this, this work proposes a “stepwise phase transition” strategy, achieving reliable fabrication of large‐area macroscopically continuous and crack‐free self‐supporting MOF glass membranes, and promoting their transition from laboratory to practical applications.

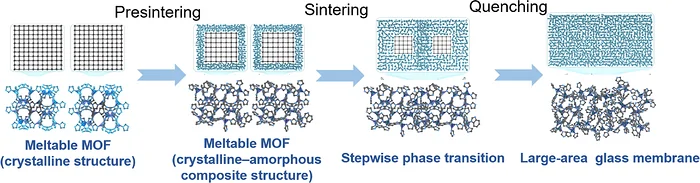

## 1 Introduction

Gas separation represents a critical enabling technology across the domains of energy transition, advanced manufacturing and healthcare [1, 2, 3]. This technique combines high permeance, low energy consumption and economic viability is desperately needed in light of the global energy restructuring and escalating climate challenges [4, 5]. Compared with conventional separation technologies, membrane‐based processes are widely regarded as sustainable and efficient solutions for industrial decarbonization, owing to their low energy requirements, minimal carbon emissions and compatibility with modular system integration [6, 7, 8]. The performance of such membrane systems is fundamentally governed by the properties of the materials employed. In recent years, metal‐organic frameworks (MOFs) have garnered substantial attention for gas separation applications. Their well‐defined pore architectures, exceptionally high surface areas and tunable chemical functionalities render them highly promising candidates for fabricating high‐performance membranes [9, 10, 11, 12, 13]. Nevertheless, most existing fabrication methods rely heavily on organic solvent‐assisted processes, which pose environmental challenges, increase production costs, and often result in membrane defects that are difficult to control [14, 15]. These drawbacks significantly hinder the scalability and practical implementation of MOF‐based membrane technologies.

Meltable MOF and MOF glass have increasingly attracted research interests as they can form a novel class of amorphous micro‐porous materials. Their unique solid‐liquid‐glass phase transitions confer exceptional solvent‐free processability and formability, generating significant research interest. Since Bennett et al. [16]. first reported the thermotropic melting of ZIF‐4, research on MOF vitrification has expanded to include various systems such as ZIF‐62 and TIF‐4 [17, 18, 19, 20]. Notably, the intrinsic meltability of these frameworks makes them particularly suitable for membrane fabrication, as the molten phase can be reshaped into continuous membranes without the constraints of solvent‐based crystal growth. Typically, the melt‐quenching fabrication of MOF glass membranes results in a uniform, dense architecture. This approach effectively circumvents the grain‐boundary defects typical of crystalline MOF membranes and eliminates the solvent‐related issues (e.g., the solvent dependency and the defects from evaporation) that plague mixed‐matrix membrane processing. This potential is demonstrated by diverse fabrication strategies for MOF glass membranes [21, 22, 23, 24, 25, 26, 27]. For example, Jiang et al. first demonstrated ZIF‐62 glass membranes for gas separation, achieving performance beyond Robeson's upper bound. Subsequently, self‐supported crystal‐glass composite (CGC) membranes and ZIF‐62 glass foam membranes were developed, delivering further improvements in gas separation performance and highlighting the versatility of MOF glass membranes as processable separation platforms. Knebel et al. [28]. recently made important progress by fabricating larger MOF glass membranes through optimized annealing and substrate selection. Nevertheless, industrial translation requires the scalable fabrication of large‐area membranes that retain high separation performance [29, 30]. In practical gas‐separation processes, the effective membrane area directly governs the gas‐processing capacity and is therefore a critical engineering parameter for industrial‐scale production. However, state‐of‐the‐art MOF glass membranes have so far been demonstrated only at relatively small scales, falling short of practical application requirements. Developing a scalable fabrication strategy for producing substantially larger, macroscopically continuous and crack‐free MOF glass membranes therefore remains an urgent priority.

Among existing fabrication routes, self‐supported MOF glass membranes are particularly attractive because they can be produced by pressing and heat treatment without relying on polymer supports or solvents, offering a potentially scalable route toward membrane production. Nevertheless, this strategy still relies on powder‐based processing and therefore inherits the intrinsic limitations of particle consolidation. Specifically, during pressing, electrostatic repulsion between charged MOF particles generates an energy barrier that hinders effective consolidation [31, 32]. This causes a fraction of the applied mechanical work to be stored as electrostatic potential energy. Upon release of the pressure, this stored energy drives elastic recovery and particle repulsion, generating microvoids and structural defects within the pressed sheet. During subsequent heat treatment, increased mobility and thermal stress further amplify these pre‐existing defects, promoting their evolution into macroscopic cracks and voids. Although such defects may be tolerable in small‐area membranes, they become increasingly detrimental during large‐area fabrication. Particle consolidation induced defects therefore represent a major barrier to the scalable production and practical deployment of high‐performance self‐supported MOF glass membranes.

Overcoming this barrier requires the large‐area membrane‐forming process to be decoupled into sequentially controlled stages rather than treated as a single vitrification step. Herein, a stepwise phase transition strategy is developed. In this design, a crystalline‐amorphous composite structure is first constructed to attenuate electrostatic repulsion between MOF particles before pressing, thereby enabling more homogeneous densification of the compact. Beyond improving particle packing, this pre‐regulated architecture provides a stabilized internal environment for the subsequent phase transition of the crystalline domains. Upon heat treatment, the amorphous component is proposed to soften and become more mobile before melting of the crystalline phase, thereby facilitating interparticle contact and the development of stronger interfacial bonding. This graded transition confines the remaining crystalline regions within a mechanically coherent matrix, suppressing defect initiation and propagation during vitrification. Consequently, large‐area, macroscopically continuous and crack‐free MOF glass membranes can be fabricated in a reliable and scalable manner.

Guided by this strategy, a high‐performance amorphous glass foam membrane with a record‐breaking area of 100 cm^2^ was fabricated, exhibiting a CH_4_ permeance of ∼13000 GPU and a CH_4_/N_2_ selectivity of ∼4.4. While the selectivity matches the similar reported values, the permeance exceeds those of previously other group reported membranes by one to two orders of magnitude. The successful assembly and validation of a single‐leaf disc‐type membrane module, with an effective area of 80 cm^2^, demonstrate its viability for practical application. Furthermore, process simulation confirms that efficient methane enrichment and recovery can be achieved with a small amount of this large‐area MOF glass membrane, underscoring its economic attractiveness. This study provides a practical technical pathway for the large‐area production of high‐performance MOF glass membranes.

## 2 Results and Discussion

Typically, meltable MOF crystals exhibit distinct phase‐transition behaviors across different temperature regimes (Figure 1a). Below the Tammann temperature, the overall framework remains structurally stable, with no obvious phase transition. Once the temperature exceeds the melting temperature(T_m_), the coordination bonds within the crystal undergo a dynamic balance of dissociation and recombination, and the crystalline long‐range ordered structure completely transforms into a liquid state. It is noteworthy that when the heating temperature is near the Tammann temperature, the coordination units at the edge regions of the MOF particles preferentially undergo dissociation and recombination, while the internal core regions still maintain a crystalline structure. After rapid quenching treatment, a composite structure consisting of both crystal and glass phases can be formed. The underlying mechanism can be interpreted from both thermodynamic and kinetic perspectives. Kinetically, as the treatment temperature approaches the Tammann regime, the mobility of coordination units in the surface region is significantly enhanced, promoting local structural relaxation and rearrangement [33]. Thermodynamically, according to the Gibbs‐Thomson effect, high‐curvature regions with elevated surface energy, such as particle edges and vertices, possess an effectively reduced melting temperature, allowing localized amorphization to occur well below the bulk melting point [34, 35]. The synergy of these two effects drives the formation of an amorphous interfacial on the crystal surface. The representative glass‐forming MOF, ZIF‐62, was selected as a model system to rigorously validate the above hypothesis. Specifically, after pre‐thermal treatment at 350°C, the sharp edges and regular morphology on the external surface of ZIF‐62 crystals were clearly diminished (Figure 1b, c), indicating localized physicochemical changes in the surface structure. This transition may stem from partial amorphization of the crystal surface. To elucidate these changes, ZIF‐62 crystals were pre‐treated at various temperatures (the resulting samples are denoted as H_X_‐ZIF‐62, where H represents to heating, X represents the heat temperature, e.g., 300°C, 350°C and 400°C) to investigate their thermal behavior. Thermogravimetric analysis (TGA) confirms negligible mass loss below 600°C for the pre‐treated samples, ruling out material decomposition as a contributing factor (Figure S3). During the first thermal cycle, H_350_ZIF‐62 exhibits an endothermic step prior to the T_m_, located near the glass transition (T_g_) step observed in the second cycle (Figure 1d), suggesting that it is not a purely crystalline phase. To gain further insight, H_400_ZIF‐62 was prepared at a higher pre‐treatment temperature. Notably, the first heating cycle of H_400_ZIF‐62 displays only a single transition near T_g_ without a discernible melting peak (Figure S4). Typically, when temperature exceeds T_m_, the crystal undergoes a transition from a long‐range ordered crystalline structure to a disordered amorphous state. The absence of a melting endotherm in the differential scanning calorimetry (DSC) curve of H_400_ZIF‐62 implies that ZIF‐62 loses most of its long‐range ordered structure after treatment at 400°C [36, 37]. This, in turn, indicates that H_350_ZIF‐62, obtained through a lower pre‐treatment temperature of 350°C, likely exists in a state where crystalline and amorphous phases coexist. To further clarify the structural evolution, in situ X‐ray diffraction patterns (XRD) measurements were conducted on ZIF‐62 crystals (Figure 1e). The diffraction intensities of ZIF‐62 decreased markedly only upon heating to 350°C, and all Bragg peaks vanished upon heating to 400°C, confirming the presence of a partial amorphous phase in H_350_ZIF‐62. This interpretation is further supported by the reduction in specific surface area and porosity (Figure 1f, Figures S5, S6 and S7), consistent with partial framework collapse and the formation of amorphous regions [38, 39]. In situ pair distribution function (PDF) analysis provided compelling evidence (Figure 1g). After heating to 350°C, H_350_ZIF‐62 retains short‐range order similar to that of the parent crystal, whereas the attenuation of oscillations at high r indicates a partial loss of long‐range order, consistent with the formation of a partially amorphous phase. In addition, the Raman and FT‐IR spectra (Figures S8 and S9) remain essentially unchanged, indicating that the local coordination environment is preserved during the pre‐heating treatment [40]. Taken together, these results demonstrate that H_350_ZIF‐62 possesses a crystalline‐amorphous composite structure.

**FIGURE 1:**
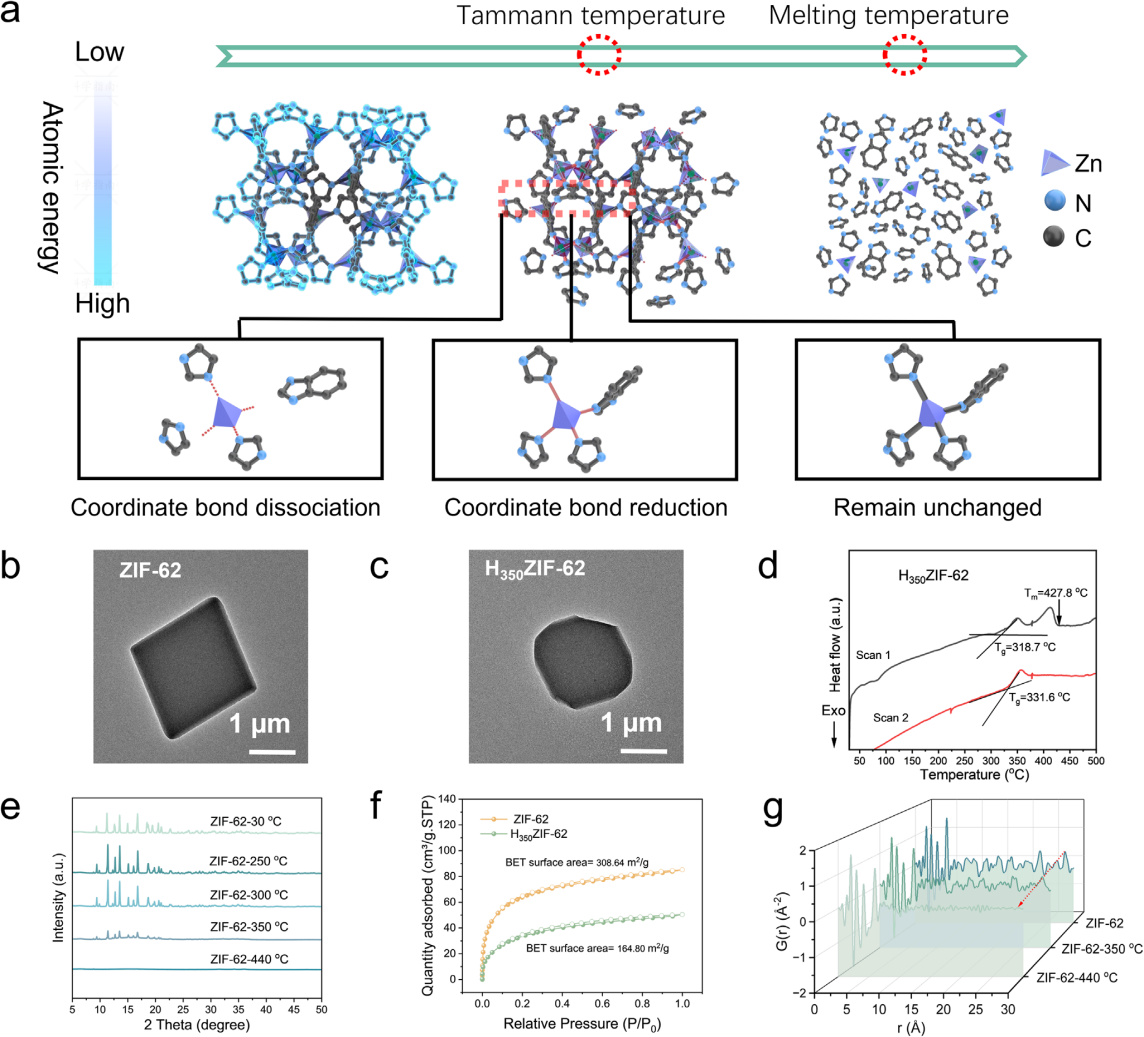
Construct MOFs with crystalline‐amorphous composite structures. (a) Structural changes of ZIF‐62 crystals under different sintering temperatures. (b) Transmission electron microscopy (TEM) image of ZIF‐62 crystals. (c) TEM image of H_350_ZIF‐62. (d) DSC curve of H_350_ZIF‐62. (e) In situ variable‐temperature PXRD patterns of ZIF‐62. (f) CO_2_ adsorption isotherms at 195 K of ZIF‐62 crystals and H_350_ZIF‐62. (g) In situ variable‐temperature PDF curves of ZIF‐62 (G(r), obtained by Fourier transform of total scattering data).

Furthermore, cyclic DSC measurements were performed on H_350_ZIF‐62 (Figure S10) to further probe the sequential thermal response of the crystalline and amorphous components. During the first heating cycle, the sample was heated to a temperature between T_g_ and T_m_ (approximately 380°C). A distinct glass transition was observed, indicating thermal activation of the pre‐existing amorphous component, while no melting endotherm was detected because the temperature remained below T_m_. During the second upscan, the sample was reheated to 480°C, which is above T_m_. An endothermic step appeared before T_m_, corresponding to the transformation of the amorphous region from the glass state to the supercooled liquid state, followed by a melting peak near T_m_, indicating that the amorphous region underwent a phase transition before the crystalline region. After cooling to room temperature again, the sample was heated for a third time to 480°C. In this cycle, only the glass transition was observed, with no detectable melting peak, consistent with the complete loss of crystallinity after the previous heating above T_m_ and subsequent vitrification. The above cyclic DSC results clearly show that the amorphous region undergoes a phase transition at a lower temperature, while the crystalline region undergoes a phase transition at a higher temperature. The coexistence of crystalline and amorphous phases leads to the stepwise phase transition behavior of the material. Figure 2 elaborates the preparation mechanism of large‐area macroscopically continuous and crack‐free MOF glass membranes driven by a stepwise phase transition. Initially, the ZIF‐62 crystals exhibit a complete long‐range ordered structure. When the temperature first rises to around the Taman temperature (approximately 350°C), the coordination units at the edges and high‐curvature regions of the crystals undergo a dissociation‐coordination dynamic equilibrium, preferentially transforming into a local liquid state, while the internal core still remains in a crystalline state. After rapid quenching, a “glassy state ‐crystalline state” composite structure (H_350_ZIF‐62) is formed. Upon reheating above the T_g_, the glass phase can show mobility, while the crystalline phase retained. At this stage, the mobility of the ligand units at the material edges significantly increases. Ligand slippage occurs first, followed by dynamic ligand interactions that promote interpenetration between adjacent particles, thereby anchoring the phase transition at the particle interfaces. When further heating above T_m_, the edge region transforms into a low‐viscosity viscous state, while the crystalline structure completely dissociates, producing a homogeneous disordered phase. This stepwise transition pathway allows the glassy state to flow and lock particle interfaces before complete melting, thereby confining the subsequent vitrification of the crystalline core and suppressing thermally induced defect formation. After controlled cooling, a large‐area, macroscopically continuous and crack‐free MOF glass membrane is obtained.

**FIGURE 2:**
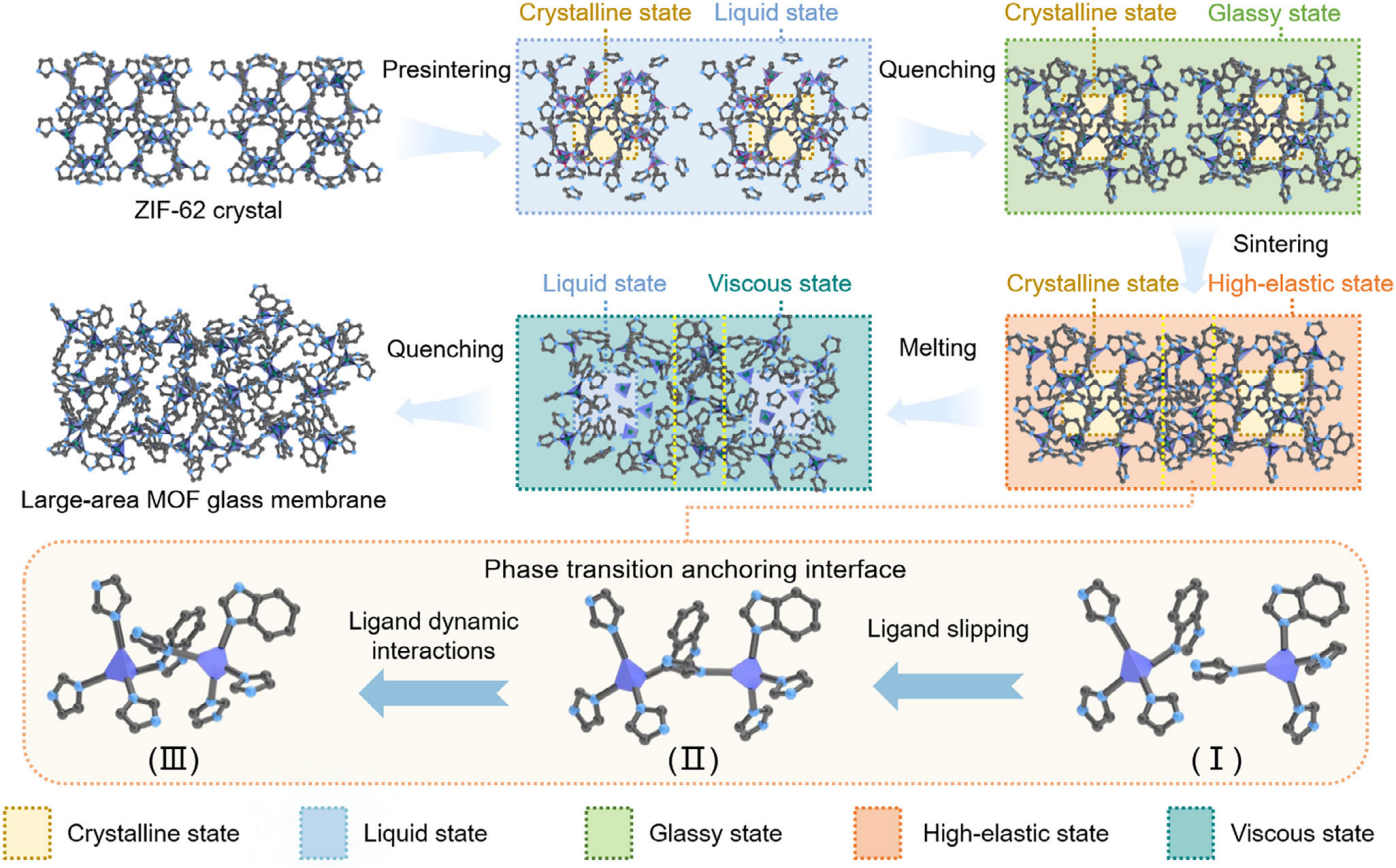
Design of a scalable fabrication strategy for MOF glass membranes. Schematic illustration of the stepwise phase transition mechanism that enables large‐area MOF glass membrane fabrication.

ZIF‐62 and H_350_ZIF‐62 powders were pressed into thin sheets under exerted pressure. Scanning electron microscopy (SEM) reveals pronounced interparticle spacing within the ZIF‐62 sheet (Figure 3a), primarily arising from strong electrostatic repulsion between particles. In contrast, the H_350_ZIF‐62 sheet shows significantly reduced interparticle spacing (Figure 3c), suggesting that pre‐thermal treatment diminishes surface electrostatic repulsion. To further validate this hypothesis, we examined the surface electrostatic interactions of the materials using Zeta potential measurements. As shown in Figure 3e, the isoelectric point (IEP) of H_350_ZIF‐62 is markedly lower than that of pristine ZIF‐62, indicating that its net surface charge approaches neutrality over a broader pH range. This shift is primarily attributed to structural rearrangements at the crystal surface during pre‐heating, which generate amorphous interfaces and substantially reduce the number of ionizable groups. Consequently, the weakened electrostatic repulsion under conventional conditions facilitates tighter particle packing. The magnitude of electrostatic force is generally determined by surface charge density [41]. Assuming an identical distance (d) between the point charge and the material surface, we measured the point potential using electrostatic force microscopy (EFM). The results reveal that H_350_ZIF‐62 exhibits a significantly lower point potential than ZIF‐62 (Figure 3f and Figure S11), directly confirming that the reduced surface charge leads to diminished electrostatic forces, thereby providing a mechanistic explanation for the tighter packing observed in H_350_ZIF‐62. After heating the pressed sheet to 350°C, the particle spacing within the ZIF‐62 sample unexpectedly increased (Figure 3b), likely due to structural relaxation triggered by enhanced thermal motion [42]. In contrast, it shows much tighter particle contacts, accompanied by locally densified regions (Figure 3d). This distinct behavior can be attributed to its crystalline‐amorphous composite structure: when reheated above its T_g_, the amorphous interfacial regions soften and undergo viscoelastic deformation ahead of the crystalline regions (Figure 3g), enabling local ligand sliding [43]. As the temperature continues to rise, adjacent particles form an interfacial interlocking structure through combined physical and chemical interactions, thereby promoting localized densification. Figure 3h and Figure S12 show surface morphologies reconstructed from AFM tip‐sample interaction data. To evaluate the mechanical properties of the interfacial region, we employed quantitative nanomechanical mapping (QNM) mode of AFM to record force‐distance curves between the probe and the sample surface, enabling determination of the Young's modulus across the interparticle transition region in the sintered compacts. Along the interfacial transition direction, the interparticle interfaces in the sintered ZIF‐62 sheet exhibit lower mechanical strength, with the Young's modulus dropping sharply as the measurement distance increases, indicating weak interparticle bonding. In contrast, the corresponding interfaces in sintered H_350_ZIF‐62 display higher overall stiffness, and the Young's modulus varies more steadily with distance. Collectively, these results consistently demonstrate that the crystalline‐amorphous composite structure effectively suppresses defect formation driven by electrostatic repulsion and thermal motion during sintering. This mechanism markedly enhances the structural stability of MOF glass membranes throughout the entire membrane‐forming process. Therefore, we successfully prepared a self‐supported MOF glass membrane with an area of 100 cm^2^ (Figure 3i).

**FIGURE 3:**
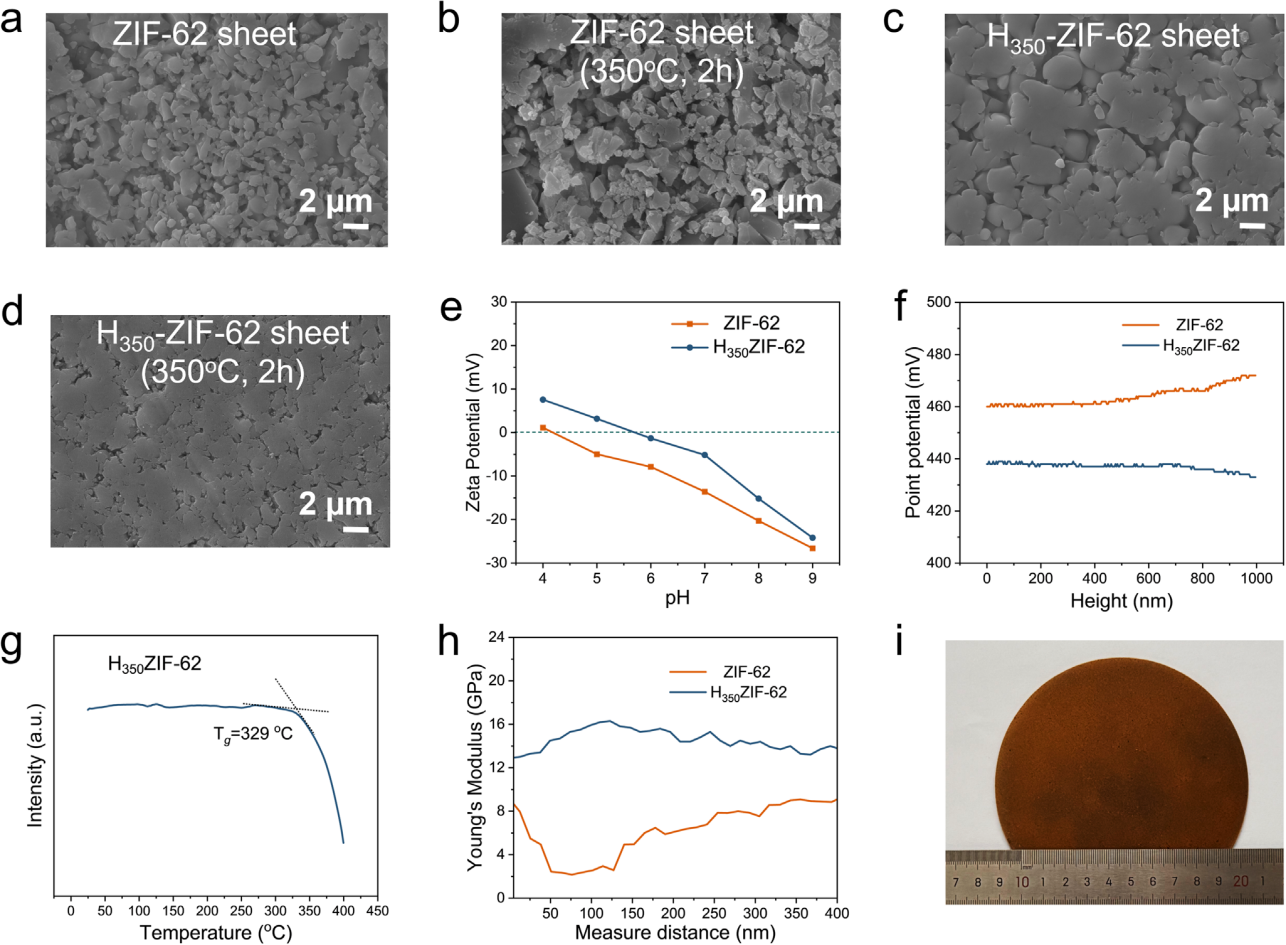
Verification of the process of assisting large‐area glass membrane formation through the stepwise phase transition process. SEM images of (a) ZIF‐62 sheet, (b) ZIF‐62 sheet after thermal treatment, (c) H_350_ZIF‐62 sheet and (d) H_350_ZIF‐62 sheet after thermal treatment. (e) Zeta potential at different pH values and (f) surface point potential comparison of ZIF‐62 and H_350_ZIF‐62 particles. (g) Thermomechanical analysis (TMA) curve of H_350_ZIF‐62. (h) Variation of the Young's modulus across the interfacial transition region between adjacent particles on the surfaces of ZIF‐62 and H_350_ZIF‐62 sheets after thermal treatment at 350°C. (i) Photographs of a_g_H_350_ZIF‐62 membranes.

Building upon the crystalline‐amorphous composite MOF materials constructed above, together with our previously reported polymer decomposition‐assisted physical pore‐forming strategy for MOF glass membranes, we systematically validated the proposed large‐area MOF glass membrane fabrication strategy (Figure 4a) [26]. Concretely speaking, the resulting H_350_ZIF‐62 powders were thoroughly mixed with PEI, pressed into sheets using molds of various dimensions, and subsequently subjected to controlled thermal treatment to yield high‐porosity, self‐supported ZIF‐62 glass‐foam membranes of corresponding sizes. The pore‐formation mechanism arises from the thermal decomposition of PEI, which releases gas (e.g., NH_3_, CO_2_ and H_2_O) during heating; the escaping gas generates a physical foaming effect that drives the formation of abundant interconnected channels [26]. This process integrates the stepwise phase transition forming process with polymer‐decomposition‐induced pore generation, thereby enabling simultaneous large‐area membrane formation and construction of a highly porous structure. As the temperature increases, the amorphous interfaces of H_350_ZIF‐62 softened and underwent viscoelastic flow in advance, enabling molecular chains from adjacent particles to contact, interpenetrate and entangle. Then, when the temperature exceeds T_m_, the long‐range ordered structure within the crystal completely disintegrates and transforms into a homogeneous liquid state. This stepwise phase transition process provided a confined and mechanically stable environment that effectively suppresses defect formation during the glass membrane forming process. Meanwhile, continued heating triggered thermal decomposition of PEI, releasing gas molecules that generated pores within the membrane via the physical pore‐forming mechanism. Ultimately, foam‐glass membranes with tunable areas and enhanced pore connectivity were successfully fabricated, demonstrating the effectiveness and scalability of the integrated membrane‐forming strategy.

**FIGURE 4:**
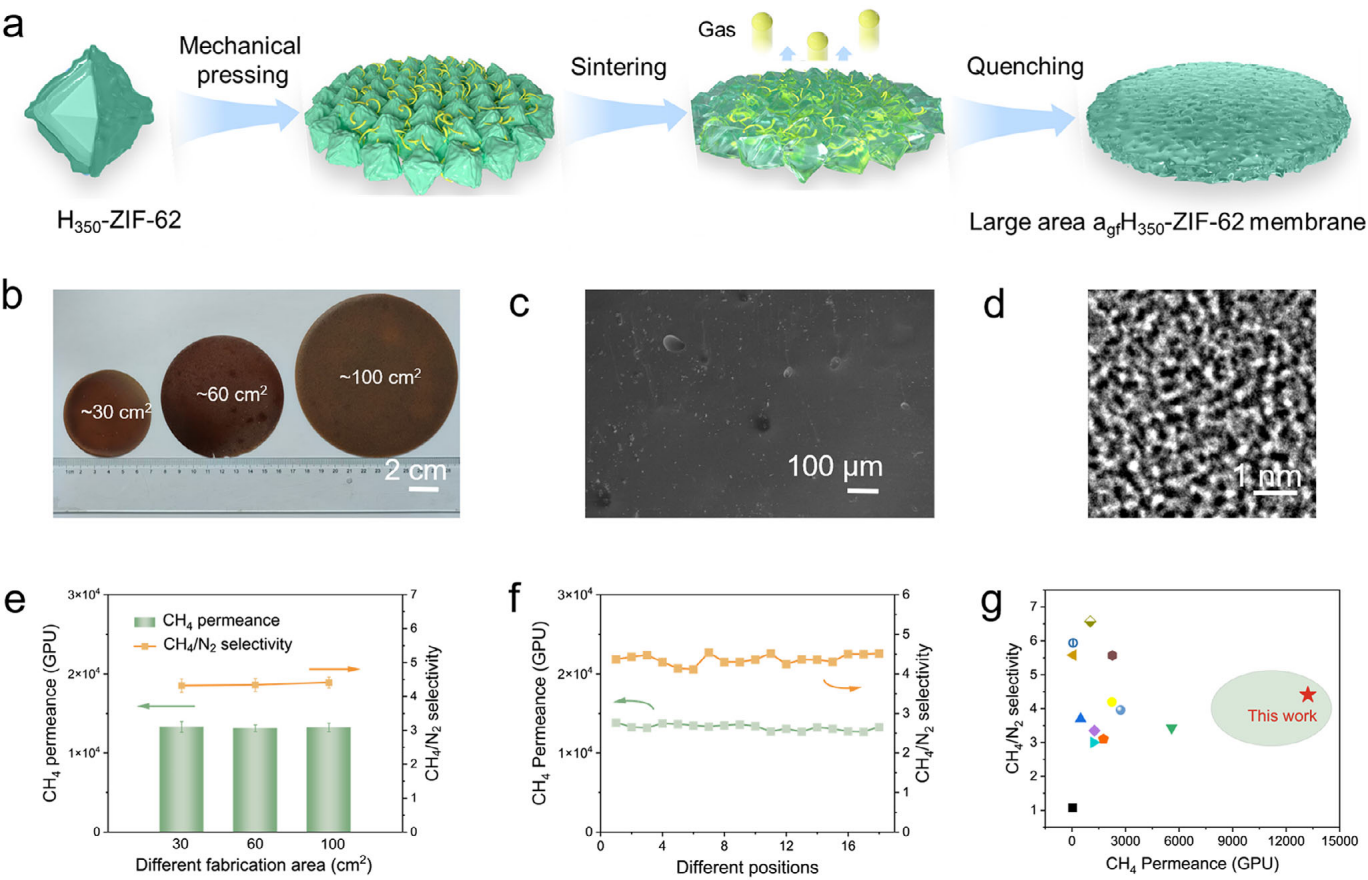
Fabrication and characterization of large‐area glass foam membranes. (a) Schematic diagram of the fabrication process for large‐area a_gf_ZIF‐62 membranes via the stepwise phase transition strategy. (b) Photographs of a_gf_H_350_ZIF‐62 membranes with different areas. (c) Cross‐section SEM images of a_gf_H_350_ZIF‐62 membrane. (d) High‐resolution TEM images of a_gf_H_350_ZIF‐62 membrane. (e) CH_4_/N_2_ separation performance of a_gf_H_350_ZIF‐62 membranes with different fabrication areas. Error bars represent the standard deviations (SD) based on three independent tests. (f) CH_4_/N_2_ separation performance for the a_gf_H_350_ZIF‐62 membranes measured at different location and (g) comparison of the CH_4_/N_2_ separation performance for the a_gf_H_350_ZIF‐62 membrane with others reported gas separation membranes.

Following this approach, uniform and dense self‐supported amorphous foam glass membranes (a_gf_H_350_ZIF‐62) with areas of 30, 60 and 100 cm^2^ were successfully fabricated, representing the first preparation of large‐scale self‐supported a_gf_H_350_ZIF‐62 membranes (Figure 4b). The transition to an amorphous glassy state was confirmed by the disappearance of diffraction peaks in XRD and the retention of characteristic peaks in FT‐IR spectra (Figures S13 and S14) [44, 45]. Membranes of varying sizes consistently demonstrated excellent mechanical performance (Figure S15 and Table S2). Evaluation of the overall quality of the a_gf_H_350_ZIF‐62 membrane (100 cm^2^) by SEM showed a generally dense surface without significant defects (Figures S16 and S17). Furthermore, to provide additional evidence of membrane uniformity, the glass membrane samples were ground and sectioned into thin slices for optical microscopy observation (Figure S18). The results further confirmed the excellent structural uniformity of the membrane at the microscopic scale. The cross‐sectional structure also exhibited uniform and dense characteristics (Figure 4c). Atomic force microscopy (AFM) further indicated a smooth and compositionally homogeneous surface, consistent with a pure glassy phase structure (Figure S19). Comparative morphological analysis revealed that the incorporation of PEI significantly increased the surface porosity of the membranes (Figure 4d, Figures S20, S21 and S22), which is attributed to pore formation by gases (e.g., NH_3_, H_2_O and CO_2_) released during PEI decomposition. TG‐MS analysis of the gaseous products from the H_350_ZIF‐62/PEI blend during heating confirmed that the main decomposition products were consistent with literature reports [26]. We conducted ^13^C MAS NMR characterization on the H_350_ZIF‐62 crystal and the a_gf_H_350_ZIF‐62 (Figure S23). The results showed that the spectra of a_gf_H_350_ZIF‐62 were basically consistent with those of the H_350_ZIF‐62 crystal, indicating that they have the same carbon chemical environment. Elemental analysis (EA) showed that the carbon content of the a_gf_H_350_ZIF‐62 membrane remained similar to the precursor H_350_ZIF‐62 (Table S3), indicating that PEI was completely removed from the membrane during the fabrication process, with no detectable residual PEI remaining.

Permselectivity represents a critical metric for gas separation membranes. To evaluate performance under practically relevant conditions, a systematic assessment of a_gf_H_350_ZIF‐62 membranes with different areas was conducted using a CH_4_/N_2_ (50 vol%/50 vol%) binary mixture at 298 K. As shown in Figure 4e, membranes fabricated with different areas consistently exhibit a CH_4_ permeance of ∼13,000 GPU with a CH_4_/N_2_ selectivity of about 4.4. Our observations indicate that the selectivity is higher than that of Knudsen diffusion (approximately 1.32), suggesting that the separation mechanism does not solely rely on Knudsen diffusion. The gas selectivity may also depend on the difference in affinity between the pore structure and various gases. We calculated the apparent activation energies of two gases using the Arrhenius equation based on the temperature‐dependent permeance rate data. The results are as follows: the apparent activation energy of CH_4_ is 0.39 kJ∙mol^−1^, and that of N_2_ is 1.04 kJ∙mol^−1^ (Figures S24, S25 and Tables S4, S5 and S6). The apparent activation energy of gas permeance reflects the combined contributions of diffusion and sorption. In the present system, the apparent activation energy of CH_4_ is lower than that of N_2_, despite the larger kinetic diameter of CH_4_. This observation suggests that molecular size‐dependent diffusion alone cannot account for the observed temperature dependence of gas permeance. Considering the adsorption isotherms, which indicate a stronger affinity of the membrane toward CH_4_ than N_2_, the lower apparent activation energy of CH_4_ is consistent with a more pronounced sorption contribution. Therefore, the combined temperature‐dependent permeance and adsorption results suggest that preferential CH_4_ sorption plays an important role in the overall CH_4_/N_2_ separation performance. And the reduced permeance compared with our previously reported glass foam membrane (∼40,000 GPU) primarily stems from the increased membrane thickness. To examine the uniform of the large‐area membrane, multiple random point tests were performed on the a_gf_H_350_ZIF‐62 membrane (100 cm^2^). The results demonstrate consistent CH_4_/N_2_ separation performance across the membrane surface (Figure 4f), indicating excellent uniformity in separation properties. These membranes exhibit gas separation performance outperforming most reported MOF‐glass and polymer‐MOF composite membranes (Figure 4g and Table S7). These results strongly validate the application potential of the stepwise phase transition strategy for the large‐scale fabrication of self‐supported glass membranes.

Membrane modules serve as fundamental units for the practical deployment of membrane technology. Leveraging their modular design, disc‐type membrane modules fully exploit the performance advantages of separation membranes while offering compact structure and small footprint, leading to their widespread application in water treatment and other industrial separation processes. Building on existing fabrication techniques, successfully prepared a single‐leaf disc‐type membrane module with an effective testing area of 80 cm^2^ (Figure 5a) and it exhibits a CH_4_ permeance of 11,584.21 GPU and a CH_4_/N_2_ selectivity of 3.97 (Figure 5b), demonstrating promising prospects for industrial application. Until now, there are no other large‐area MOF glass membrane successfully prepared. To further evaluate the potential of this large‐area MOF glass foam membrane for methane enrichment, process simulations were conducted for a typical methane recovery system with a feed gas of 1000 kmol/h (Figure 5c) [46]. The simulation results indicate that a two‐stage membrane process employing our MOF glass foam membrane can achieve efficient methane enrichment and recovery. Only a minimal amount of membrane is needed to raise the methane concentration from 50% to 90% (Figure 5d)—a purity level that meets the basic requirements for subsequent resource utilization. Notably, while maintaining high product purity, the membrane system achieves a methane recovery rate exceeding 90%, highlighting its significant advantage in industrial gas separation: ensuring product quality while minimizing feed gas loss, thereby substantially enhancing the overall process economic competitiveness. Furthermore, the module maintains outstanding CH_4_/N_2_ separation performance after 100 h of continuous operation, as well as under different testing temperatures and elevated‐pressure conditions (Figures 5e, f and g), exhibiting remarkable chemical and structural stability. This behavior can be attributed to the structural stability of the MOF glass framework. Within the investigated pressure range, the applied pressure is insufficient to induce appreciable framework deformation or pore compression. Likewise, over the examined temperature range, the relatively low activation barriers for CH_4_ and N_2_ diffusion through the ultramicropores result in only a modest temperature dependence of their transport rates. Consequently, the membrane maintains nearly unchanged gas separation performance under variations in pressure and temperature. Therefore, the developed MOF glass membrane module and its stepwise phase transition strategy demonstrate significant potential for industrial applications.

**FIGURE 5:**
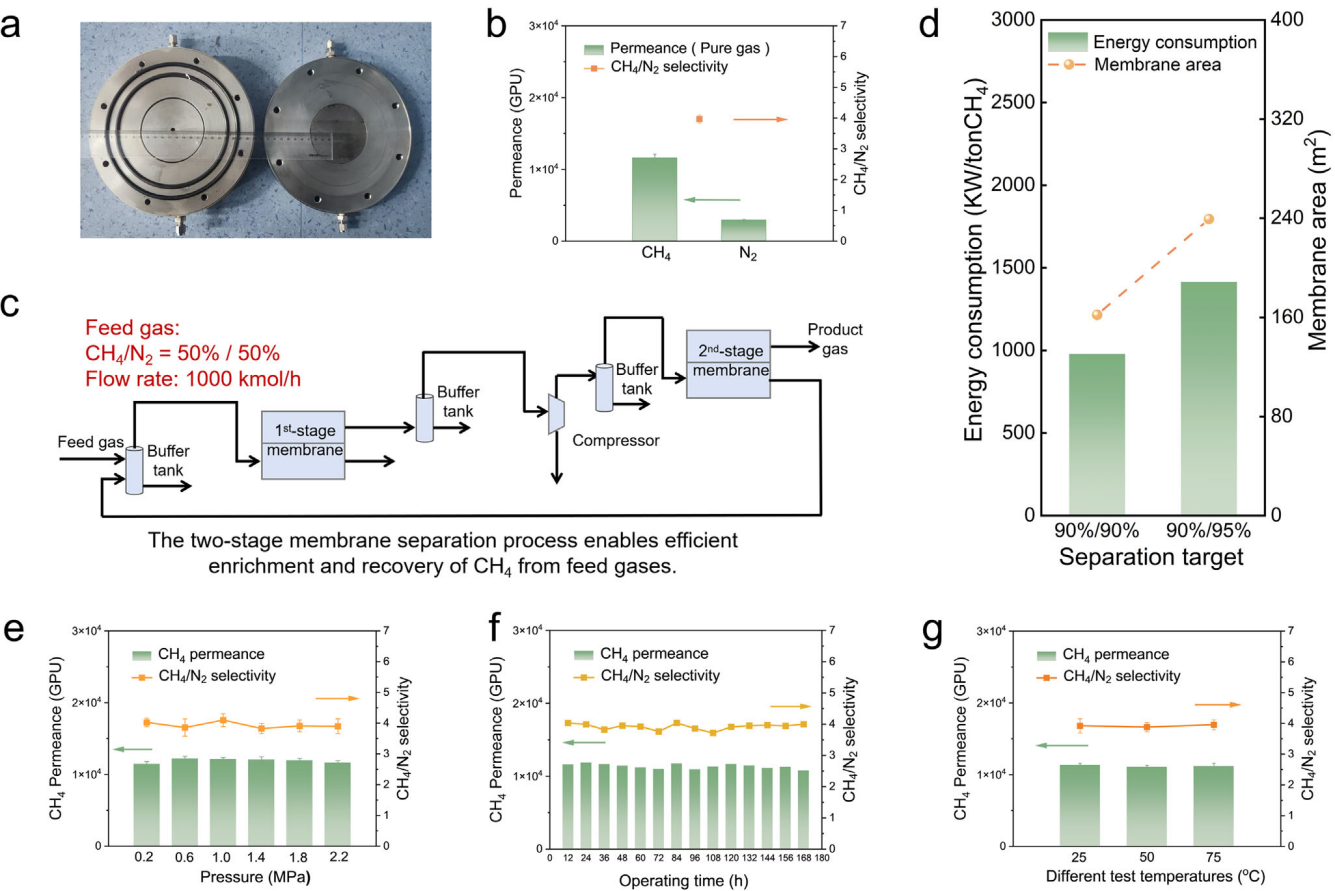
Evaluation of CH_4_/N_2_ separation performance and process‐level of large‐area a_gf_H_350_ZIF‐62 modules. (a) Schematic illustration of a single‐leaf disc‐type membrane module with an effective testing area of 80 cm^2^. (b) CH_4_/N_2_ separation performance of the membrane module. (c) Illustration of the two‐stage membrane separation process for the methane recovery system (Simulation conditions: the feed gas flow rate is 1000 kmol/h, the feed gas pressure is 2.0 MPa, the composition is 50 vol% N_2_ and 50 vol% CH_4_). (d) Energy consumption and membrane area required to achieve the dual 90% CH_4_ purity/recovery rate or higher separation target. CH_4_/N_2_ separation performance for the membrane module measured (e) during long‐term operation, (f) under different testing pressures and (g) at different testing temperatures. Error bars represent the SD based on three independent tests.

## 3 Conclusion

In summary, this work introduces an innovative “stepwise phase transition” strategy to overcome defect propagation during the fabrication of large‐area MOF glass membranes. This approach systematically mitigates the key failure mechanisms associated with interparticle electrostatic repulsion and unconstrained thermal motion of molecular chains. The strategy exploits the sequential kinetic behavior of crystalline‐amorphous composite MOF materials. A preformed amorphous interface mitigates interparticle repulsion during pressing, enabling efficient densification of the powder compact. Upon heating, the amorphous regions flow viscously prior to the crystalline regions, facilitating early interface interlocking that creates a confined, stable environment for uniform vitrification of the crystalline regions phase. Based on this strategy, self‐supported a_gf_H_350_ZIF‐62 membranes with an area of 100 cm^2^ were successfully fabricated, representing a substantial advancement in scalability. These membranes exhibit stable and superior CH_4_/N_2_ separation performance. The successful development of a disc‐type module, combined with process‐level assessment, confirms its potential in applications such as methane enrichment. Overall, this work provides a practical route for fabricating large‐area, high‐performance MOF glass membranes and establishes a new paradigm for managing non‐equilibrium phase transitions in complex powder‐based systems. Meanwhile, this strategy offers a generalizable methodology for the controlled fabrication of advanced powder‐derived materials. It marks a critical step toward the practical deployment of MOF glasses and lays a strategic foundation for scaling up next‐generation functional materials for sustainable separation technologies.

## Author Contributions

**Mao Ye**: conceptualization, methodology, data curation, validation, investigation, Writing – original draft, Writing – review and editing. **Yi Yang**: conceptualization, methodology, validation, investigation, writing – original draft, writing – review and editing. **Zibo Yang**: conceptualization, methodology, data curation, investigation, validation, writing – original draft, writing – review and editing. **Yuxiu Sun**: conceptualization, methodology, writing – review and editing, writing – original draft, data curation. **De Ao**: conceptualization, methodology. **Fan Zhou**: methodology. **Ying Chen**: data curation. **Chunhui Luo**: investigation. **Ziyao Wang**: conceptualization. **Wenjia Sun**: validation. **Yunling Jia**: data curation. **Wengang Huang**: supervision, project administration, writing – review and editing, writing – original draft, funding acquisition, conceptualization, methodology. **Jingwei Hou**: conceptualization, methodology, funding acquisition, project administration, writing – original draft, writing – review and editing, supervision. **Zhi Wang**: conceptualization, methodology, writing – original draft, writing – review and editing, funding acquisition, project administration, supervision. **Zhihua Qiao**: conceptualization, methodology, writing – original draft, writing – review and editing, project administration, funding acquisition, supervision.

## Conflicts of Interest

The authors declare no conflicts of interest.

## Supporting information




**Supporting File**: advs78073‐sup‐0001‐SuppMat.docx.

## Data Availability

The data that support the findings of this study are available from the corresponding author upon reasonable request.
