# Supplementary material for: Scalable Fabrication of MOF Glass Membranes for Gas Separation via Stepwise Phase Transition
Source: Adv Sci (Weinh). 2026 Sep 29:e78073. Online ahead of print. doi: 10.1002/advs.78073 (PMC13624317; doi:10.1002/advs.78073)
Supplement: Supplementary file 1 [file ADVS-9999-e78073-s001.docx]

Supporting Information

Scalable fabrication of MOF glass membranes for gas separation via stepwise phase transition

Mao Ye^†[a b]^, Yi Yang^†[c]^, Zibo Yang^†[e]^, Yuxiu Sun^†[a]^, De Ao^[f]^, Fan Zhou^[a]^, Ying Chen^[a^ ^b]^, Chunhui Luo^[a]^, Ziyao Wang^[a]^, Wenjia Sun^[a]^, Yunling Jia^[a]^, Wengang Huang*^[d, g]^, Jingwei Hou*^[d g]^, Zhi Wang*^[c]^, Zhihua Qiao*^[a]^

[a]	Mao Ye, Yuxiu Sun, Fan Zhou, Ying Chen, Chunhui Luo, Ziyao Wang,,Wenjia Sun, Yunling Jia and Zhihua Qiao*
State Key Laboratory of Advanced Separation Membrane Materials
Tiangong University
Tianjin 300387, China
E-mail: qiaozhihua@tiangong.edu.cn

[b]	Ying Chen, Mao Ye
School of Textile Science and Engineering
Tiangong University
Tianjin 300387, China

[c]	Yi Yang, Zhi Wang
School of Chemical Engineering and Technology
Tianjin University
Tianjin 300350, China
E-mail: wangzhi@tju.edu.cn

[d]	Wengang Huang, Jingwei Hou
School of Chemical Engineering
The University of Queensland St Lucia
QLD 4072, Australia
E-mail: [Wengang.huang@uq.edu.au](mailto:Wengang.huang@uq.edu.au), [Jingwei.hou@uq.edu.au](mailto:Jingwei.hou@uq.edu.au).

[e]	Zibo Yang
Hebei Center for New Inorganic Optoelectronic Nanomaterial Research
Handan University
Handan, Hebei 056005, China

[f]	De Ao
School of Materials Science and Engineering
Inner Mongolia University of Science and Technology
Baotou, 014010, China

[g]	Wengang Huang, Jingwei Hou
ARC Centre of Excellence for Green Electrochemical Transformation of Carbon Dioxide
Brisbane 4072, Australia

^†^These authors contributed equally to this work. *Corresponding authors

This PDF file includes:

Experimental Section

Figures S1 to S25

Tables S1 to S7

References

Materials and Methods

**Materials**

The following reagents were of analytical grade and used as received: imidazole (Im, 99%) was purchased from Anhui Senrise Technology Co., Ltd (China), benzimidazole (bIm, 98.5%) was purchased from Macklin Biochemical Technology Co., Ltd. (China), zinc nitrate hexahydrate (Zn(NO_3_)_2_ 6H_2_O, 99%) was purchased from Aladdin Reagent Co., Ltd. (China), polyethyleneimine, (PEI, Mw=300) was purchased from Shandong Xiya Chemical Co., Ltd (China), dimethylformamide (DMF) and methanol were purchased from Sinopharm Chemical Reagent Co., Ltd. (China).

**Methods**

**Synthesis of ZIF-62**

ZIF-62 was synthesized following a previously reported procedure with minor adjustments (*S1*). Briefly, Zn(NO_3_)_2_·6H_2_O (1.65 g, 5.54 mmol) and imidazole (Im, 8.91 g, 131 mmol) were dissolved in 50 mL of DMF, while benzimidazole (bIm, 1.55 g, 13.1 mmol) was separately dissolved in 25 mL of DMF. The two solutions were combined under vigorous stirring to form a homogeneous mixture, which was subsequently transferred to a 100 mL Teflon-lined autoclave and heated at 130 ^o^C for 48 h. After cooling to room temperature, the solid product was collected by centrifugation and thoroughly washed several times with DMF followed by methanol. The obtained solid was finally activated under vacuum at 150 ^o^C for 24 h to yield crystalline ZIF-62.

**Preparation of H_X_ZIF-62.**

ZIF-62 crystals were thermally treated in a tube furnace for 2 h at the designated temperature, and the resulting samples were named as H_X_ZIF-62, where X represents the treatment temperature.

**Fabrication of H_350_ZIF-62 amorphous glass membranes (a_g_H_350_ZIF-62).**

To prepare the glass membrane, a predetermined amount of H_350_ZIF-62 powder was mixed with methanol in an agate mortar, with the volume of solvents and mass of solids adjusted according to the targeted membrane area. The mixture was thoroughly ground to ensure uniform dispersion. The resulting slurry was transferred into pellet die with diameters of 13 cm and evenly distributed. A uniaxial pressure of 15 MPa was then applied for 60 s to form sheets, which were carefully transferred onto glass slides. Subsequently, the sheets were heated in a tube furnace under a nitrogen atmosphere. The temperature was ramped to 440 ^o^C at a rate of 3 ^o^C min^-1^ and maintained for 30 min. After natural cooling to room temperature, the final amorphous glass membranes (a_g_H_350_ZIF-62) were obtained.

**Fabrication of H_350_ZIF-62 amorphous glass-foam membranes (a_gf_H_350_ZIF-62).**

To prepare the glass foam membranes (*26*), a predetermined amount of H_350_ZIF-62 powder was mixed with methanol and a polyethyleneimine (PEI) solution in an agate mortar, with the volume of solvents and mass of solids adjusted according to the targeted membrane area. The mixture was thoroughly ground to ensure uniform dispersion. The resulting slurry was transferred into pellet dies with diameters of 7, 9, or 13 cm and evenly distributed. A uniaxial pressure of 15 MPa was then applied for 60 s to for sheets, which were carefully transferred onto glass slides. Subsequently, the sheets were heated in a tube furnace under a nitrogen atmosphere. The temperature was ramped to 440 ^o^C at a rate of 3 ^o^C min^-1^ and maintained for 30 min. After natural cooling to room temperature, the final amorphous glass foam membranes (a_gf_H_350_ZIF-62) were obtained.

**Characterization**

High-resolution transmission electron microscopy (HR-TEM, Jeol JEM-F200, Japan) was employed to characterize the structural evolution of ZIF-62 before and after thermal treatment, as well as the pore features on the surface of the resulting a_gf_H_350_ZIF-62 membranes.

Thermogravimetric analysis (TGA, Netzsch 209F3 Tarsus, Germany) was conducted to examine the mass-change behavior of the materials at different temperatures. Differential scanning calorimetry (DSC, Netzsch 200 F3, Germany) was used to determine the melting temperature and glass-transition temperature.

X-ray diffraction (XRD, Bruker D2 PHASER, Germany) was used at room temperature to analyze the crystallinity of the materials. In situ XRD measurements under controlled heating were performed to monitor the structural evolution of ZIF-62 as a function of temperature.

CO_2_ adsorption isotherms at 195 K were measured using a surface-area and porosity analyzer (BET, BSD-660, China), and the pore-size distributions were derived using the non-local density functional theory (NLDFT) model. For validation, N_2_ adsorption isotherms at 77 K and Ar adsorption isotherms at 87 K were also collected.

X-ray total scattering data of ZIF-62 at various temperatures were collected in situ at the PD beamline of the *Australian Nuclear Science and Technology Organization* (ANSTO) using a Mythen-II detector. The maximum momentum transfer, Qmax, was set to 18 Å^-1^ to ensure high-quality S(Q) data. The obtained S(Q) profiles were Fourier-transformed and refined using the PDFgetX3 software to generate the corresponding pair-distribution functions G(r), providing insights into the local structural evolution.

Fourier-transform infrared spectroscopy (FT-IR, Bruker 1244 ATR-IR, Germany) and Raman spectroscopy (Horiba HR Evolution, Germany) were employed to analyze the molecular structures and chemical-bond variations of ZIF-62 in different states.

Atomic force microscopy (AFM, Bruker Dimension ICON, Germany) was employed to characterize the surface morphology of the a_gf_H_350_ZIF-62 membranes and to analyze the surface potential of pressed ZIF-62 and H_350_ZIF-62 samples under identical conditions. The electrical-property distribution was further examined using the electrostatic force microscopy (EFM) mode, in which the intermittent contact mode detects variations in electrostatic interactions with the sample surface, providing information on local surface potential and charge distribution. After thermal treatment of the pressed samples at 350 ^o^C, by using the quantitative nanomechanical mapping (QNM) mode via the force-distance curves between the probe and the sample surface were recorded to determine the Young’s modulus. During these measurements, the probe approached and retracted from the surface at a constant speed while the corresponding force response was continuously monitored.

Scanning electron microscopy (SEM, Carl·Zeiss Gemini500, Germany) was used to examine the morphological evolution during membrane formation and to determine the membrane thickness. A dynamic light scattering and zeta potential analyzer (DLS, Malvern Zetasizer Nano ZS90, UK) was employed to measure the surface charge characteristics of ZIF-62 and H_350_ZIF-62 under different pH conditions.

Thermomechanical analysis (TMA, Netzsch 402 F3 Hyperion^®^, Germany) was performed to monitor the thermal expansion and softening behavior of the samples as a function of temperature. Measurements were conducted under a nitrogen atmosphere in compression mode, with a preload of 0.1 N applied to ensure proper contact between the sample and the platform. The temperature was increased from room temperature to 400 ^o^C at a heating rate of 10 ^o^C min^-1^.

TGA was performed on Netzsch STA449F3 with parallel online MS analysis (Bruker, QMS403C Germany) at a heating rate of 3 ^o^C min^-1^ from 20 to 440 ^o^C and holding for 20 min, following a cooling procedure with a rate of 5 ^o^C min^-1^ from 440 ^o^C to 30 ^o^C. The process was undertaken in an Ar atmosphere with a flow rate of 100 mL min^-1^.

The ^13^C cross-polarization magic-angle spinning (CP-MAS) nuclear magnetic resonance spectrum of the sample was obtained using a solid-state nuclear magnetic resonance spectrometer (Avance Neo 600M, Bruker, Germany) The obtained spectrogram is used to analyze the chemical environment of carbon in the membrane material, in order to verify whether PEI has been completely removed during the heat treatment process.

The content of each element was determined by using the high-temperature combustion method with an elemental analyzer (EA, Elementar Vario EL). The content of the sample was studied based on the content of these elements. The sample was dried in a vacuum oven at 120 °C for 12 hours.

The adsorption and separation capabilities of the membrane material for CH_4/_N_2_ at 298K were analyzed using the fully automatic gas adsorption analyzer (ASAP 2020 Plus HD88, Micromeritics).

The mechanical properties of the membranes were tested using a nanoindentation instrument (Hysitron TI 950). On the polished glass sample, based on the load-displacement data (with a load of 10 mN), the average hardness (H) and Young's modulus (E) were recorded, and five measurements were conducted at each position.

**Gas permeance measurements**

The gas-separation performance of the a_gf_H_350_ZIF-62 membranes was evaluated using a constant-pressure/variable-volume setup (Figure S1). A dried binary gas mixture (components *i* and *j,* flow rate: 500 mL/min) was fed into the upstream side of the membrane cell. Under an applied pressure gradient, a portion of the gas permeated through the membrane and reached the downstream side, while the non-permeating fraction exited through the upstream outlet. During the gas testing process, a concentration polarization phenomenon occurs, resulting in errors in the separation performance. The main reason for this phenomenon is that the concentration of the test gas below the membrane is higher than that above the membrane. Eventually, due to the concentration difference, reverse diffusion occurs. Therefore, we use He as the purge gas to reduce the concentration of the test gas below the membrane, thereby reducing this effect. At the same time, He is a commonly used carrier gas in gas chromatography (GC), and using He as the purge gas can directly be compatible with the GC carrier gas system without the need for additional gas switching or correction steps. He (flow rate: 200 mL/min) was used as the carrier gas to sweep the permeate stream into a gas chromatograph (GC, Agilent 8890, America) for compositional analysis. The gas permeance was calculated from the molar flow rate, pressure difference, and membrane area according to the following equation:

$R_{i}=\frac{Q_{i}}{S*\Delta P_{i}}$	(1)

where *R_i_* is the permeance of component *i* (in GPU; 1 GPU=10^-6^ cm^3^ (STP) cm^-2^ s^-1^·cmHg^-1^), *Q_i_* is the molar flow rate of component *i* (cm^3^ (STP) s^-1^), *S* is the membrane area (cm^2^), and *ΔP_i_* is the transmembrane pressure difference of component i (Pa, 1 Pa = 7.5*10^-4^ cmHg).

The separation selectivity of component *i* over component *j* was determined from the ratio of their permeances, given by:

$\alpha_{i/j}=\frac{R_{i}}{R_{j}}$	(2)

**Techno-economic evaluation of the large area MOF glass foam membranes fabricated in this work.**

The technical and economic evaluation of the MOF glass foam membranes using a two-stage membrane process was adopted to evaluate the large-scale application potential in methane enrichment fled. Specifically, the implementation of the technical and economic evaluation is computed by Pro/Ⅱ software according to previous work (*46*).

For a typical methane recovery process, the feed gas flow rate is 1000 kmol/h, the feed gas pressure is 2.0 MPa, and the composition is 50 vol% CH_4_ and 50 vol% N_2_. During the simulation, the feed gas pressure of 1^st^-stage and 2^nd^-stage is maintained at 2.0MPa to calculate the specific energy consumption required to achieve the recovery target and the total membrane area required to achieve the processing capacity and specific energy consumption.

Supplementary Figures

**Figure S1.** (**a) stainless steel pressing mold (non-standard size) used for pressing the diaphragm and (b) the inorganic glass base used to support and transfer the samples during the heat treatment process.**

**Figure S2. Schematic illustration of the constant-pressure membrane separation setup used for gas permeance measurements.**

**Figure S3. TGA curves of (a) ZIF-62 and (b) H_350_ZIF-62 upon heating.** Both materials exhibit negligible mass loss over below 600  ^o^C, indicating excellent thermal stability. This confirms that the thermal events observed in the DSC measurements do not arise from material decomposition.

**Figure S4. DSC curves of H_400_ZIF-62.** Upon increasing the prethermal-treatment temperature to 400 ^o^C, the endothermic melting peak of ZIF-62 disappears, while a distinct endothermic step emerges in the glass-transition region.

**Figure S5. Pore size distribution calculated by NLDFT method of ZIF-62 crystals and H_350_ZIF-62. Data source: CO_2_ adsorption isotherms of ZIF-62 crystal and H_350_ZIF-62 at 195 K.**

**Figure S6. (a) Ar adsorption isotherms at 87 K and (b) Pore size distribution calculated by NLDFT method of ZIF-62 crystals and H_350_ZIF-62.**

**Figure S7. (a) N_2_ adsorption isotherms at 77 K and (b) NLDFT pore size distributions of ZIF-62 and H_350_ZIF-62.**

**Figure S8. Raman spectra of ZIF-62 crystals and H_350_ZIF-62.**

**Figure S9. FT-IR spectra of ZIF-62 crystals and H_350_ZIF-62.**

**Figure S10.The cyclic DSC curve of H_350_ZIF-62.**

**Figure S11. EFM data of (a) ZIF-62 and (b) H_350_ZIF-62.** Both the surface potential and phase-shift signals of H_350_ZIF-62 are noticeably lower than those of ZIF-62, which is attributed to molecular rearrangements occurring on the crystal surface during thermal treatment.

**Figure S12. AFM topography of (a) H_350_ZIF-62 and (b) ZIF-62 nanosheets after thermal treatment at 350 ^o^C.** The arrow indicates the measurement direction of the Young’s modulus.

**Figure S13. XRD patterns of H_350_ZIF-62 and a_gf_H_350_ZIF-62.** After the melt-quench membrane-forming process, the characteristic diffraction peaks of H_350_ZIF-62 disappear and are replaced by a broad amorphous peak. This transformation results from the dynamic breaking and re-formation of Zn-N coordination bonds when the material is heated to 440 ^o^C, leading to the progressive loss of the long-range periodic order present in H_350_ZIF-62. Upon quenching, the material solidifies into an amorphous glass that retains only short-range structural order.

**Figure S14. FT-IR spectra of H_350_ZIF-62 and a_gf_H_350_ZIF-62.** After the melt-quench process, the characteristic absorption peaks of H_350_ZIF-62 remain essentially unchanged. This is because, although the material undergoes a transition from long-range crystalline order to short-range ordered glass, the local coordination environment is largely preserved during melting and quenching.

**Figure S15. Load-displacement curves of a_gf_H_350_ZIF-62 membranes with different fabrication areas.**

**Figure S16. High-magnification SEM images of the a_gf_H_350_ZIF-62 membrane taken at different surface locations.**

**Figure S17. Low-magnification SEM images taken from different regions of the a_gf_H_350_ZIF-62 membrane.** The uniformly dense surface observed over large areas confirms the structural integrity of the membrane, while the high consistency among different locations demonstrates the stability and reproducibility of the fabrication process.

**Figure S18. Optical microscope images of the a_gf_H_350_ZIF-62 membrane taken at different surface locations.**

**Figure S19. AFM phase diagram of a_gf_H_350_ZIF-62 membrane.**

**Figure S20. HR-TEM image of a_gf_H_350_ZIF-62.**

**Figure S21. HR-TEM images of the a_gf_H_350_ZIF-62 membrane.** HR-TEM images collected from randomly selected regions of the a_gf_H_350_ZIF-62 membrane confirm the presence of a well-developed pore structure.

**Figure S22. TGA-MS profiles of the H_350_ZIF-62/PEI mixture during heating, showing the release of NH_3_, H_2_O and CO_2_ originating from the thermal decomposition of PEI.**

**Figure S23. ^13^C MAS NMR spectra of H_350_ZIF-62 and a_gf_H_350_ZIF-62.**

**Figure S24. (a) CH_4_/N_2_ separation performance for the a_gf_H_350_ZIF-62 membrane measured at different testing temperature and (b) Adsorption isotherms of a_gf_ZIF-62 for CH_4_ and N_2_ at 298 K.**

**Figure S25. Arrhenius temperature dependency of a_gf_H_350_ZIF-62 membranes.**

Supplementary Tables

**Table S1.** Configuration of GC8890

| **Symbol** | **model number** |
| --- | --- |
| Thermal conductivity detector | G3540A 220 |
| 6-way valve | G3540A 701 |
| 10-way valve | G3540A 801 |
| Chromatographic column | G3591-81020 |
| Chromatographic column | G3591-8101 |

**Table S2.** Hardness (H) and Young's modulus (E) of a_gf_H_350_ZIF-62 membranes with different fabrication areas.

|  | 30 cm^2^ | 60 cm^2^ | 100 cm^2^ |
| --- | --- | --- | --- |
| H | 0.716616 | 0.838705 | 0.848161 |
| E | 7.96984 | 7.952837 | 7.923403 |

The units of H and E are GPa.

**Table S3.** Elemental Analysis

| **Symbol** | **C** | **N** | **H** |
| --- | --- | --- | --- |
| H_350_ZIF-62 | 40.23% | 25.84% | 3.11% |
| a_gf_H_350_ZIF-62 | 40.17% | 25.65% | 3.08% |

**Table S4.** The permeance of CH_4_ through the a_gf_H_350_ZIF-62 membrane within the temperature range from 298 K to 348 K.

| **T** | **T** | **P (GPU)** |
| --- | --- | --- |
| 25 | 298 | 12987.38 |
| 35 | 308 | 13136.09 |
| 45 | 318 | 12954.34 |
| 55 | 328 | 13070.00 |
| 65 | 338 | 13251.765 |
| 75 | 348 | 13317.85 |

**Table S5.** The permeance (P) of N_2_ through the a_gf_H_350_ZIF-62 membrane within the temperature range from 298 K to 348 K.

| **T** | **T** | **P (GPU)** |
| --- | --- | --- |
| 25 | 298 | 2891.26 |
| 35 | 308 | 2856.76 |
| 45 | 318 | 2937.26 |
| 55 | 328 | 2960.26 |
| 65 | 338 | 3017.77 |
| 75 | 348 | 3040.78 |

**Table S6.** Permeance activation energies of CH_4_ and N_2_ for a_gf_H_350_ZIF-62 membranes. The temperature dependence of the permeance follow an Arrhenius relationship (*S2*):

$$P=P_{0}exp(\frac{{-E}_{act}}{RT})$$

Where E_act_ is permeance activation energies (kJ mol^-1^), P is the gas permeance, P_0_ is the pre-exponential factors. R is the theoretical gas constant (8.314 J∙mol^-1^∙K^-1^), T is the test temperature (K).

| **Gas** | **E_act_(kJ·mol^-1^ )** |
| --- | --- |
| CH_4_ | 0.39 |
| N_2_ | 1.04 |

**Table S7.** A comparison of the CH_4_/N_2_ separation performance of the a_gf_H_350_ZIF-62 membrane with that of the reported gas separation membranes.

| **Symbol** | **Membrane** | **CH_4_ permeance**  **(GPU)** | **CH_4_/N_2_ selectivity** | **Ref** |
| --- | --- | --- | --- | --- |
|  | ZIF-62-Br-based MMMs | 2235 | 4.2 | *S3* |
|  | CoNi-DABCO@PDMS/PVDF MMMs | 484 | 3.7 | *S4* |
|  | PVA/ACU-120A/MPSf MMMs | 5584 | 3.43 | *S5* |
|  | PVAm/SRZ/MPSf MMMs | 1261 | 3.35 | *S6* |
|  | VdZIF Membrane | 60.8 | 5.58 | *S7* |
|  | TPC-RCC3@PIP | 1216 | 3 | *S8* |
|  | PVAm/(SCH-M)_0.7_(SCH-Z)_0.3_ MMMs | 2263.94 | 5.57 | *S9* |
|  | Honeycomb-like MOF MMMs | 1025 | 6.57 | *S10* |
|  | ZIF-8 MMMs | 1763 | 3.1 | *S11* |
|  | ZIF-62-L/PEI MMMs | 2726.6 | 3.96 | *S12* |
|  | ZIF-8 membrane | 35.4 | 1.07 | *S13* |
|  | GZIF_m10_ | 64.9 | 5.94 | *S14* |
|  | a_gf_H_350_ZIF-62 membrane | 13217.88 | 4.40818 | *This work* |

References

[S1]	S. Li, R. Limbach, L. Longley, A. A. Shirzadi, J. C. Walmsley, D. N. Johnstone, P. A. Midgley, L. Wondraczek, T. D. Bennett, “Mechanical properties and processing techniques of bulk metal-organic framework glasses,” *Journal of the American Chemical Society* 141, no. 3 (2019): 1027-1034. <https://doi.org/10.26434/chemrxiv.7234826.v1>

[S2]	P. Hülsmann, K. A. Weiß, M. Köhl, “Temperature-dependent water vapour and oxygen permeation through different polymeric materials used in photovoltaic-modules,” Progress in Photovoltaics: Research and Applications 22, no. 4 (2014): 415-421. <https://doi.org/10.1002/pip.2273>

[S3]	C. Ma, N. Li, W. Xue, X. Guo, Z. Qiao, C. Zhong, “Polarization enhanced CH_4_/N_2_ separation in bromine functionalized ZIF-62 based mixed-matrix membranes,” *Journal of Membrane Science* 683 (2023): 121829. <https://doi.org/10.1016/j.memsci.2023.121829>

[S4]	J. Wang, Y. Ying, P. Guo, D. Liu, W. Zhang, Q. Yang, “Fabrication of Hofmann-type metal-organic framework based mixed-matrix membranes for efficient CH_4_/N_2_ separation,” *Journal of Membrane Science* 713 (2025): 123387. <https://doi.org/10.1016/j.memsci.2024.123387>

[S5]	Z. Yu, Z. Gu, J. Lei, G. Zheng, “Vacuum treated amorphous MOF mixed matrix membrane for methane/nitrogen separation,” *Journal of Solid State Chemistry* 320 (2023): 123852. <https://doi.org/10.1016/j.jssc.2023.123852>

[S6]	Z. Gu, X. Zhang, R. Liang, F. Wang, D. Wang, A. Chen, Z. Qiao, “Large-Area Ultrathin Mixed Matrix Membranes Incorporating Small Rigid MOFs for Gas Separation,” *ACS Applied Polymer Materials* 7 (2025): 11890-11900. <https://doi.org/10.1021/acsapm.5c02357>

[S7]	X. Huang, Y. Xiao, H. Guo, W. Li, “Ultrathin node‐missing glass membranes enabled by vapor deposition of lattice-defective ZIF nanofilms for gas separation,” *Small* 21 no. 43 (2025): e08431. <https://doi.org/10.1002/smll.202508431>

[S8]	Z. Jiang, Y. Wang, M. Sheng, Z. Zha, J. Wang, Z. Wang, S. Zhao, “A highly permeable porous organic cage composite membrane for gas separation,” *Journal of Materials Chemistry A* 11, no. 13 (2023): 6831-6841. <https://doi.org/10.1039/d2ta09632c>

[S9]	R. Liang, L. Wang, Y. Wang, F. Zhou, Z. Yang, Y. Sun, Z. Gu, Z. Qiao, “High-vacuum-calcined multi-MOF mixed-matrix membrane for CH_4_/N_2_ separation,” *Polymer* 309 (2024): 127455. <https://doi.org/10.1016/j.polymer.2024.127455>

[S10]	Y. Liang, C. Yu, X. Yang, Z. Qiao, “Preparation of ultrathin and highly loaded MOF mixed matrix membranes with honeycomb-like structure via ordered array self-assembly,” *Chemical Engineering Journal* 485 (2024): 149749. [https://doi.org/10.1016/j.cej.2024.149749](%20https:/doi.org/10.1016/j.cej.2024.149749)

[S11]	Z. Gu, Z. Yang, X. Guo, Z. Qiao, C. Zhong, “Vacuum resistance treated ZIF-8 mixed matrix membrane for effective CH_4_/N_2_ separation,” *Separation and Purification Technology* 272 (2021): 118845. <https://doi.org/10.1016/j.seppur.2021.118845>

[S12]	C. Ma, Z. Yang, X. Guo, Z. Qiao, C. Zhong, “Size-reduced low-crystallinity ZIF-62 for the preparation of mixed-matrix membranes for CH_4_/N_2_ separation,” *Journal of Membrane Science* 663 (2022): 121069. <https://doi.org/10.1016/j.memsci.2022.121069>

[S13]	Y. S. Li, F. Y. Liang, H. Bux, A. Feldhoff, W. S. Yang, J. Caro, “Molecular sieve membrane: Supported metal-organic framework with high hydrogen selectivity,” *Angewandte Chemie International Edition* 49, no. 3 (2010): 548-551. <https://doi.org/10.1002/anie.200905645>

[S14]	Y. Xiao, Y. Yu, X. Huang, D. Chen, W. Li, “Directly Gel-Thermal Processing of Linker-Mixed Crystal-Glass Composite Membranes for Sorption-Preferential Gas Separation,” *Advanced Science* 12 (2024): 2413942. <https://doi.org/10.1002/advs.202413942>
